# Performance of the Vitek 2, Sensititre, and Etest antimicrobial susceptibility testing systems for isolates of *Burkholderia cepacia* complex

**DOI:** 10.1128/jcm.00484-25

**Published:** 2026-01-15

**Authors:** Kileen L. Shier, Giovanna Gonzales, Dece Ann G. Perez, Jian R. Bao, Kamran N. Azad

**Affiliations:** 1 Quest Diagnostics 828715 , Chantilly, Virginia, USA; 2 Quest Diagnostics 355082 , San Juan Capistrano, California, USA; Vanderbilt University Medical Center, Nashville, Tennessee, USA

**Keywords:** Vitek 2, antimicrobial susceptibility testing, *Burkholderia cepacia*, Etest, Sensititre

## Abstract

*Burkholderia cepacia* complex (BCC) is a highly antimicrobial-resistant bacteria that frequently colonizes patients with cystic fibrosis. Previous studies have demonstrated inconsistent performance and questionable reliability of disk diffusion, agar dilution, gradient diffusion strip, and Microscan methods. This study evaluated the performance of the Biomerieux Vitek 2, Thermo Scientific Sensititre broth microdilution, and Etest methods for susceptibility testing of BCC organisms in comparison to a previously characterized isolate set. Of the three methods tested, Sensititre had the highest Essential and Categorical agreements (EA and CA, respectively) , though it still produced multiple Very Major Errors (VME). Vitek 2 had the lowest EA and CA, especially for ceftazidime and meropenem. For all three methods, there was a trend of lower minimum inhibitory concentrations and increased Susceptible results in comparison to the reference broth microdilution method, resulting in a high number of VME. While none of the methods met acceptance criteria, Sensititre may offer the best commercially available option for clinical laboratories in circumstances where antimicrobial susceptibility testing is warranted.

**IMPORTANCE:**

Antimicrobial susceptibility testing (AST) of *Burkholderia cepacia* complex isolates remains of interest due to their inherent multidrug resistance. Previous studies have demonstrated unreliable performance of various methods. As clinical laboratories seek options to perform testing in cases where AST is needed, this study provides data on the performance of three commercially available methods: the Biomerieux Vitek 2, Thermo Scientific Sensititre panels, and the Biomerieux Etest.

## INTRODUCTION

*Burkholderia cepacia* complex (BCC) are gram-negative (GN) bacilli known for their drug resistance and colonization of patients with cystic fibrosis (CF), though they can also cause respiratory and bloodstream infections in other patients. Antimicrobial susceptibility testing (AST) is often requested both for patient management and eligibility determination for lung transplants in cystic fibrosis patients (1).

The Clinical Laboratory Standards Institute (CLSI) removed disk diffusion (2) and minimum inhibitory concentration (MIC) breakpoints (3) from the Performance Standards for AST due to previously reported MIC inconsistencies between disk diffusion, agar dilution, and reference broth microdilution (rBMD) methods (1, 4, 5). The European Committee on Antimicrobial Susceptibility Testing (EUCAST) does not recommend routine AST for BCC (6). A previous study demonstrated a poor correlation of MICs obtained with the Microscan to rBMD (1); however, no data were published on the widely used Vitek 2 system.

While the CLSI method for BCC AST is rBMD, frozen broth microdilution panels are not commercially available. Thermo Scientific Sensititre panels offer a commercially available lyophilized broth microdilution AST system that may be the closest method to rBMD.

The aim of this study was to evaluate the performance of Biomerieux Vitek 2 GN AST panels and a Thermo Scientific Sensititre panel for BCC isolates. Etest gradient diffusion strips were included due to their common use as an alternative to automated AST methods.

## MATERIALS AND METHODS

A validation set of BCC isolates was obtained from the University of Michigan Cystic Fibrosis Foundation *Burkholderia cepacia* Laboratory and Repository (UMCFFBCLR) containing 46 isolates from both CF (*n* = 24) and non-CF patients (*n* = 22) with various sources (sputum *n* = 28, blood *n* = 11, and other sources *n* = 7). The isolates had been characterized for their modal MICs and interpretations using the CLSI rBMD method at two laboratories, Clinical Microbiology Institute and Vanderbilt University Medical Center (3–6 replicates per isolate). The UMCFFBCLR shipped the isolates at −80°C in cryotubes with Lysogeny Broth (LB) and glycerol to the Quest Diagnostics, Chantilly, VA laboratory (CHY). For Sensititre and Etest, the isolates were subcultured to nutrient agar slants from the frozen stocks received from the University of Michigan, incubated overnight at 36°C–38°C, and then shipped ambient to Quest Diagnostics, San Juan Capistrano, CA (SJC), where frozen stocks were prepared.

In the CHY laboratory, isolates were subcultured from the frozen stocks received from the UMCFFBCLR onto Remel sheep blood agar plates (Lenexa, KS) twice prior to testing. The Biomerieux Vitek 2 (Durham, NC) was performed using both a gram-negative (GN) AST-802 (ceftazidime [CAZ], levofloxacin [LVX], meropenem [MEM], trimethoprim-sulfamethoxazole [TMP-SMX] and a GN AST -XN15 (minocycline, MIN) card for all isolates. Briefly, a suspension from isolated colonies on the second passage of 18–24 h growth on a Remel sheep blood agar plate was prepared equivalent to a 0.5 McFarland standard in Biomerieux saline and loaded onto a Vitek 2 XL instrument using version 9.02 and Observa software.

For testing by Sensititre (Thermo Fisher, Oakwood Village, OH) in the SJC laboratory, a subculture on a Remel sheep blood agar plate was prepared from the frozen stocks prepared in-house and then subcultured a second time prior to preparation of a 0.5 McFarland equivalent suspension in 5 mL Sensititre demineralized water from colonies less than 24 h old. Thirty microliters was transferred into 11 mL Sensititre cation-adjusted Muller Hinton broth. The tube was then mixed prior to transferring 50 mL into each well of the Sensititre GN7F broth microdilution panel within 15 min. Isolates were incubated at 34°C–36°C in ambient air for 20–24 h and read manually according to the manufacturer’s instructions.

To perform the Etest (Biomerieux, Durham, NC) in the SJC laboratory, isolates from colonies less than 24 h old grown on Remel sheep blood agar plates were used to prepare a 0.5 McFarland equivalent suspension in saline (Remel). The suspensions were streaked onto a Mueller-Hinton Agar (Remel) in three directions, and an Etest strip was plated within 15 min of streaking. Plates were incubated at 35°C–37°C in ambient air for 18–20 h. MICs were read manually according to the manufacturer’s instructions.

Isolates with discrepant MIC results, defined as greater than ± 1 doubling dilution from those provided by the UMCFFBCLR, and all isolates that terminated prematurely were repeated, and the repeat results are reported in the tables. Concordant results were not repeated unless the isolates were selected for precision testing. Categorical interpretations were based on the CLSI 2024 Performance Standards for AST (2). There is no Intermediate interpretation for TMP-SMX and BCC. Essential (EA) and categorical agreement (CA), along with very major errors (VME), major errors (ME), and minor errors (mE) were calculated according to the definitions by CLSI (7). Acceptance was defined as ≥ 90% for EA and CA, ≤ 1 VME and ME combined due to the small number of isolates, and < 10% mEs.

## RESULTS

For the 46 BCC isolates on Vitek 2 GN AST panels, EA ranged from 44.2% to 95.2% and CA ranged from 58.1 to 92.9%, with only TMP-SMX meeting EA and CA acceptance criteria (Table 1). There were nine VMEs for MEM (47.4%), eight for CAZ (38.1%), three for TMP-SMX (17.6%), and one each for MIN (8.3%) and LVX (5.0%). There were ten mEs for CAZ and LVX (22.7%), nine for MEM (20.9%), and eight for MIN (19.0%), none of which met acceptance criteria. There was one ME for MIN (4.0%). In addition, there were 2–4 terminated results for each antimicrobial agent on the Vitek panels. Two isolates terminated for all five antimicrobial agents analyzed, including repeated testing (Tables S1 to S5). No antimicrobials met all requirements for acceptability.

**TABLE 1:** Performance of the Vitek 2 AST system for 46 isolates^*a*^

Antimicrobial		EA	CA	VMEs	MEs	mEs	TRM
CAZ	Number	24/44	26/44	8/21	0/23	10/44	2/46
	Percent	54.5%	59.1%	38.1%	0.0%	22.7%	4.3%
LVX	Number	38/44	33/44	1/20	0/19	10/44	2/46
	Percent	86.4%	75.0%	5.0%	0.0%	22.7%	4.3%
MEM	Number	19/43	25/43	9/19	0/19	9/43	3/46
	Percent	44.2%	58.1%	47.4%	0.0%	20.9%	6.5%
MIN	Number	30/42	32/42	1/12	1/25	8/42	4/46
	Percent	71.4%	76.2%	8.3%	4.0%	19.0%	8.7%
TMP-SMX	Number	40/42	39/42	3/17	0/25	0/42	4/46
	Percent	95.2%	92.9%	17.6%	0.0%	0.0%	8.7%

^*a*^ CA = Categorical Agreement; EA = Essential Agreement; MEs = Major Errors; mEs = Minor Errors; TRM = termination; VMEs = Very Major Errors. Terminated results were excluded from EA, CA, VMEs, MEs, and mEs.

Due to concerns about the reproducibility of automated AST systems for BCC, reproducibility of the Vitek 2 system was evaluated. Three randomly selected isolates were tested in triplicate on 1 day and in singlet on 2 additional days. All antimicrobial agents had consistent MIC results (100% EA, Table S6) but not all met EA when compared to the reference results.

Forty-five isolates were tested by the Sensititre method. One isolate was nonviable after shipment. EA ranged from 84.4% to 97.8% and CA ranged from 68.9% to 88.9% (Table 2), with EA being acceptable for CAZ (97.8%) and LVX (93.3%). There were six VMEs for TMP-SMX (28.6%); two each for LVX (9.1%), MEM (9.5%), and MIN (12.5%); and one for CAZ (4.5%). There were 12 mEs for MEM (26.7%), eight for MIN (17.8%), seven for LVX (15.6%), and four for CAZ (8.9%). No MEs were observed. CAZ met all of the acceptance criteria with the exception of CA, which was close at 88.9%. No other antimicrobials met all of the acceptance criteria.

**TABLE 2:** Performance of the Sensititre AST system for 45 isolates^*a*^

Antimicrobial		EA	CA	VMEs	MEs	mEs
CAZ	Number	44/45	40/45	1/22	0/23	4/45
	Percent	97.8%	88.9%	4.5%	0.0%	8.9%
LVX	Number	42/45	36/45	2/22	0/19	7/45
	Percent	93.3%	80.0%	9.1%	0.0%	15.6%
MEM	Number	40/45	31/45	2/21	0/19	12/45
	Percent	88.9%	68.9%	9.5%	0.0%	26.7%
MIN	Number	38/45	35/45	2/16	0/25	8/45
	Percent	84.4%	77.8%	12.5%	0.0%	17.8%
TMP-SMX	Number	39/45	37/45	6/21	0/25	0/45
	Percent	86.7%	82.2%	28.6%	0.0%	0.0%

^*a*^ CA = Categorical Agreement; EA = Essential Agreement; MEs = Major Errors; mEs = Minor Errors; VMEs = Very Major Errors.

The same 45 isolates were also tested by Etest. EA ranged from 48.9% to 93.3%, and CA ranged from 77.3% to 88.9% (Table 3), with EA acceptable for LVX (93.3%) and MEM (91.1%) and CA unacceptable for all agents, although CAZ was again close at 88.9%. There were seven VMEs for TMP-SMX (33.3%), three for MIN (18.8%), two for MEM (9.5%), and one each for CAZ (4.5%) and LVX (4.5%). There were nine mEs for LVX (20.0%), seven each for MEM (15.6%) and MIN (15.6%), and five for CAZ (11.1%), all unacceptable. No MEs were observed.

**TABLE 3:** Performance of the Etest AST system for 45 isolates^*a*^

Antimicrobial		EA	CA	VMEs	MEs	mEs
CAZ	Number	32/45	40/45	1/22	0/23	5/45
	Percent	71.1%	88.9%	4.5%	0.0%	11.1%
LVX	Number	42/45	34/45	1/22	0/19	9/45
	Percent	93.3%	77.3%	4.5%	0.0%	20.0%
MEM	Number	41/45	36/45	2/21	0/19	7/45
	Percent	91.1%	81.8%	9.5%	0.0%	15.6%
MIN	Number	33/45	35/45	3/16	0/25	7/45
	Percent	73.3%	77.8%	18.8%	0.0%	15.6%
TMP-SMX	Number	22/45	38/45	7/21	0/25	0/45
	Percent	48.9%	86.4%	33.3%	0.0%	0.0%

^*a*^ CA = Categorical Agreement; EA = Essential Agreement; MEs = Major Errors; mEs = Minor Errors; VMEs = Very Major Errors.

The categorical interpretations varied widely compared to the reference data (Tables S1 to S5), with more Intermediate results when tested with Vitek 2 in comparison to the reference method. All three methods (Vitek 2, Sensititre, and Etest) had more Susceptible interpretations in comparison to rBMD, indicating a trend of lower MICs with the test methods in comparison to rBMD.

## DISCUSSION

AST in BCC organisms has previously shown to be inconsistent, but interest in AST remains due to multidrug resistance and eligibility determination for lung transplantation in CF patients. Our data further document the high variability in MIC results among widely used AST methods. 

Of the three test systems, Thermo Scientific Sensititre had the best performance, with EA ranging from 84.4% to 97.8% and CA ranging from 68.2% to 88.9%. VMEs were most often observed for TMP-SMX (six), though two of the six VMEs were within a doubling dilution of a Resistant result. LVX, MEM, and MIN had a higher minor error rate than is typically considered acceptable, though the number of isolates was small (45 isolates). None of the antimicrobials tested met acceptance criteria for EA, CA, and error rates. Of the methods tested, Sensititre plates are most similar to rBMD, with the primary difference being commercial preparation of standardized microdilution plates with lyophilized antimicrobial agents as opposed to manual preparation of rBMD in the laboratory. The shared methodology of broth microdilution may explain why Sensititre performed best in comparison to rBMD.

Etest had excellent EA for LVX (93.3%) and MEM (91.1%), but EA for TMP-SMX was low at 48.9%. CA was low for all antimicrobials tested (77.3%–88.9%). None of the seven TMP-SMX VMEs were within a doubling dilution of a Resistant interpretation. All other antimicrobials had an elevated mE rate (11.1%–20.0%). These findings are consistent with those of previous studies showing near-acceptable performance with LVX by Etest but unacceptable performance for other antimicrobial agents, though MEM performed better in our study than previously reported (4, 5). Importantly, BCC is not included in the Etest indication, and our study supports that Etest is not a reliable method for BCC AST.

Vitek 2 had the worst performance of the systems tested. EA, CA, and VMEs were all poor for CAZ and MEM. MIN performed only slightly better. Interestingly, the performance was best for TMP-SMX, with acceptable EA and CA. While the TMP-SMX EA and CA did not meet the acceptance criteria for Sensititre, Vitek 2 had only 1 more isolate with EA and 2 more isolates with CA than did Sensititre.

There are multiple limitations to our study. First, the isolate set was relatively small. Second, isolates had to be shipped to a second location for two of the testing methodologies, introducing the risk of loss of resistance. One isolate was nonviable after shipment. Third, while rBMD has demonstrated acceptable intra-assay reproducibility, it does not correlate with other methods, and therefore the true MICs remain unknown.

While *Burkholderia cepacia* is listed in the package insert as a claimed organism for the Vitek 2 AST cards, the high rates of both terminations and VMEs from our results suggest this organism might not grow adequately on the Vitek 2 AST panels, leading to potentially unreliable AST results from the microdilution-based Vitek 2 AST system. The Thermo Scientific Sensititre system performed better than Vitek 2 and was the closest to meeting the standard acceptance criteria for an AST method. Etest continues to demonstrate suboptimal performance. In the absence of a fully reliable method to perform BCC AST, clinical microbiology laboratories may consider utilizing Sensititre panels in cases where BCC AST is required.

## SUPPLEMENTAL MATERIAL

Tables S1 to S6 jcm.00484-25-s0001.xlsx Detailed results.

ASM does not own the copyrights to Supplemental Material that may be linked to, or accessed through, an article. The authors have granted ASM a non-exclusive, world-wide license to publish the Supplemental Material files. Please contact the corresponding author directly for reuse.
